# Height From the Ground and Phytotelm pH Affect Prokaryotic Diversity in Epiphytic Bromeliads

**DOI:** 10.1111/1758-2229.70421

**Published:** 2026-10-22

**Authors:** Xaali O'Reilly‐Berkeley, Thomas D. Hughes, Hannah Mossman, Jennifer K. Rowntree, Richard F. Preziosi

**Affiliations:** ^1^ Departament de Biologia Animal, de Biologia Vegetal i d'Ecologia Universitat Autònoma de Barcelona Bellaterra Spain; ^2^ CREAF Bellaterra Spain; ^3^ Department of Natural Sciences The Manchester Metropolitan University Manchester UK; ^4^ Timburi Cocha Research Station San José de Payamino Orellana Ecuador; ^5^ Instituto Nacional de Biodiversidad Quito Pichincha Ecuador; ^6^ School of Biological and Marine Sciences University of Plymouth Plymouth UK

**Keywords:** bromeliad, eDNA, metabarcoding, phytotelm, prokaryotic diversity

## Abstract

Tank bromeliads in the neotropics harbour complex communities of invertebrates and even provide food, water and shelter to various vertebrate species. However, few studies have sequenced the microbial communities within epiphytic bromeliad phytotelmata. We sequenced the 16S rRNA subunit extracted from swabs rubbed at the base within the central phytotelm of the epiphytic bromeliads *Aechmea hoppii* and *A. nidularioides*. We tested factors that we suspected could influence the alpha diversity and beta‐diversity of prokaryotic communities in bromeliads. Height on the tree and size of the bromeliad influenced the alpha‐diversity within plants. Height from the ground and pH of the phytotelm explained small proportions of the variation among communities, regardless of whether quantitative or qualitative distances were calculated. We used FAPROTAX to assign taxa to functional groups and found that a complex picture of correlations between function and variables emerged, with height on the tree, pH, plant size and species, forest type and sampling season correlating with different functional groups. This relatively large metabarcoding study of bromeliad phytotelmata prokaryotes contributes to the growing knowledge of these highly diverse and complex systems.

## Graphical abstract

The prokaryotic community composition within epiphytic bromeliad phytotelmata is influenced by habitat and plant characteristics, such as pH, volume and the height at which the plant is found. In addition, various features correlate with different functional groups of prokaryotes within the bromeliad phytotelm.

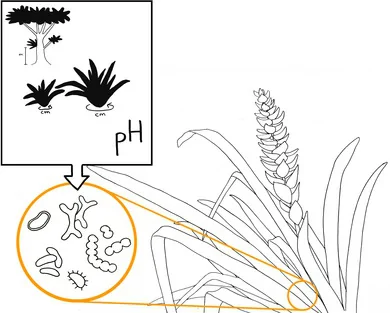

## 1 Introduction

Knowledge of the microbial diversity in the tropical understory and canopy is limited (Nakamura et al. 2017), but there have been some notable studies exploring this diversity on suspended soils (Donald et al. 2020; Eskov et al. 2021), leaf surfaces (Lambais et al. 2006; Kembel et al. 2014) and phytotelmata (Pittl et al. 2010; Gilbert et al. 2020). Bromeliads form important phytotelmata in neotropical habitats, including in megadiverse forests such as Amazonia. These plants form phytotelmata by virtue of their spiral rosette structure, which allows for rainwater to collect between their leaf axils. The phytotelmata are colonised by diverse communities of eukaryotes and prokaryotes. Eukaryotic microorganisms in bromeliads include rotifers, ciliates, tardigrades, yeasts and crustaceans, among others (Gomes et al. 2015; Simão et al. 2017; Mercado‐Salas et al. 2021). Prokaryotic microorganisms (Bacteria and Archaea) in bromeliads have been shown to be incredibly diverse, with previous studies identifying between 23 and 51 different phyla in a few samples collected within close proximity of each other (Rodriguez‐Nuñez et al. 2018; Herrera‐García et al. 2022). Although there are similarities in the microbial communities among individual bromeliads from the same species (Louca, Jacques, Pires, et al. 2017), there is still a high degree of variation in community structure among individuals (Carmo et al. 2014; Louca, Jacques, Pires, Leal, Srivastava, et al. 2017) and even between separate phytotelmata within the same bromeliad (Giongo et al. 2019), emphasising a role for environmental and stochastic processes in structuring these communities (Farjalla et al. 2012).

Prokaryotes perform a wide range of functions in bromeliad phytotelmata, from breaking down plant and animal detritus (Goffredi, Jang, et al. 2011), methane production and nitrogen cycling (Inselsbacher et al. 2007; Goffredi, Kantor, et al. 2011; Kotowska and Werner 2013), to primary production (Lehours et al. 2016). These functions benefit both the plants themselves as well as their associated eukaryotic communities by providing important nutrients to these microcosms (Inselsbacher et al. 2007; Gonçalves et al. 2014; Leroy et al. 2016).

There have been various approaches to studying the prokaryotic communities in bromeliads. Non‐DNA‐based methods have included studying bacterial growth or chemical production (Haubrich et al. 2009; Martinson et al. 2010; Brandt et al. 2017), as well as flow cytometry and staining, which are useful for counting microorganisms and identifying them based on morphology without the need to culture them. Despite the benefits of these approaches, they have low taxonomic resolution and it is often unfeasible to obtain full coverage of complex communities. DNA‐based approaches such as DGGE electrophoresis have allowed the discrimination of taxonomic richness and abundance based on size and intensity of amplified DNA fragments (Farjalla et al. 2012; Céréghino et al. 2020) while DNA metabarcoding with high‐throughput sequencing has enabled an analysis of whole microbial communities at greater taxonomic resolution, based on a barcode or barcodes of choice (Rodriguez‐Nuñez et al. 2018). However, there are still relatively few studies that have used next‐generation sequencing to characterise bromeliad‐associated communities, although they are now increasing in number, with at least 10 publications on prokaryote communities to date (Louca, Jacques, Pires, Leal, González, et al. 2017; Louca, Jacques, Pires, Leal, Srivastava, et al. 2017; Rodriguez‐Nuñez et al. 2018; Giongo et al. 2019; Simão et al. 2020; Aguilar‐Cruz et al. 2022; Herrera‐García et al. 2022; Hakobyan et al. 2023; Huber et al. 2025). However, such studies generally target bromeliads on or close to the ground, leaving epiphytic bromeliad prokaryotic communities largely unexplored.

Bromeliad phytotelmata house distinct prokaryotic communities compared to their surroundings (Hakobyan et al. 2023; Huber et al. 2025). Several biogeochemical factors have been shown to influence prokaryotic communities in bromeliads. For instance, phytotelmata pH, ammonium ion concentration, water temperature, water colour and turbidity and methane concentrations (Haubrich et al. 2009; Goffredi, Kantor, et al. 2011; Louca, Jacques, Pires, Leal, Srivastava, et al. 2017), as well as algal biomass and tank depth (Brouard et al. 2011; Goffredi, Jang, et al. 2011) have been shown to be related to prokaryotic taxonomic or functional diversity. Additionally, some external environmental factors may affect these prokaryotic communities, such as the presence or absence of surrounding vegetation or habitat type, canopy cover and detrital content (Louca, Jacques, Pires, Leal, Srivastava, et al. 2017; Aguilar‐Cruz et al. 2022; Herrera‐García et al. 2022). Overall, attachment height on the host tree has been neglected due to most of these studies focusing on bromeliads within reach from the ground.

In this study, we looked at how habitat and physical plant characteristics influence the prokaryotic phytotelm communities of two epiphytic bromeliads in one of the most biodiverse regions on Earth, the Tropical Andes Biodiversity Hotspot. We collected data from two vegetatively cryptic species of bromeliads, which were later identified using inflorescences and molecular methods. We characterised the prokaryote communities by sequencing a section of the 16S rRNA subunit and add to growing body of literature on bromeliad community metabarcoding. We analysed the effects of forest regeneration stage (primary or secondary), height on the host tree, pH, size of the plant, complexity of the plant, as well as the bromeliad species. Based on the existing literature on prokaryotic bromeliad communities, we expected factors such as the pH and forest type to affect composition (Louca, Jacques, Pires, Leal, Srivastava, et al. 2017; Aguilar‐Cruz et al. 2022; Hakobyan et al. 2023), while the large body of research on bromeliad invertebrates suggests that bromeliad size has an impact on communities (Armbruster et al. 2002; Srivastava et al. 2008; Jocque and Field 2014). Although there is little research on the effect of height on these communities, height of the attachment of the bromeliad on the host tree was shown to explain a small part of variation in invertebrate communities within these same bromeliads (O'Reilly‐Berkeley et al. 2026) and so we expected this to be mirrored in the composition of prokaryotic taxa and functional groups, due to components of these being trophically linked (Céréghino et al. 2020).

## 2 Experimental Procedures

### 2.1 Focal Species

Two species of congeneric bromeliad were selected in this study, *Aechmea hoppii* (Harms) (L.B. Smith 1953a) and *A. nidularioides* (L.B. Smith 1953b). They were chosen based on their abundance in the field site and are physically indistinguishable when no inflorescence is present. The clustering of plants based on distances calculated with specially‐designed microsatellite markers (Table S1) was used to separate the two species, as this clustering coincided with the separation of plants by their inflorescences. The microsatellite development is described in the Supporting Information, as well as the distinction between the species of bromeliad in the sample (Figure S1).

### 2.2 Field Sampling

This study was carried out in San José de Payamino, Orellana, Ecuador, from here on referred to as Payamino. Researchers were based at the Timburi Cocha Research station in Payamino 0°28′57.9″ S, 77°17′05.1″ W. The site is described in detail O'Reilly‐Berkeley et al. (2026), but briefly, Payamino is a 17,000 ha patchwork of primary and secondary at various successional stages, as well as traditional farmlands. It is owned by the indigenous community of San José de Payamino whose permission and help was essential to carry out the study. Field sampling took place in January–February and July–August 2018. Although Payamino does not have marked seasons (Irvine 1987; Sullivan et al. 2020), sampling period was included as a factor in all analyses.

This project was carried out in accordance with the formal agreement between the San José de Payamino Community and the Timburi Cocha Research Station and with permission from the Ministry of the Environment, Water and Ecological Transition of Ecuador (Ministerio de Ambiente Agua y Transición Ecológica MAATE) under the access to genetic resources research permits MAE‐DNB‐CM‐2016‐0045 and MAE‐DNB‐CM‐2019‐0121.

Bromeliads were collected between 1.96 and 20.20 m off the ground, using either a ladder or double‐rope canopy access techniques. The height of the bromeliad was determined by dropping the end of a tape measure to the ground, while holding the top against the base of the plant. Whether the site a bromeliad was found in was primary or secondary forest was determined by the author and the expertise of the local guides.

Upon accessing a bromeliad, a sterile swab was rubbed for 10–20 s at the bottom of the base of the central phytotelm and immediately placed in a sterile 2.0 cryovial. Samples were frozen in liquid nitrogen upon return to the base camp that day. This method was used to standardise sampling between bromeliads, as the water level varied between bromeliads and it was not possible to store larger vials in the basic living conditions of the research station.

Following swabbing, a handheld pH metre was used to record pH and temperature of the central phytotelm. The bromeliad was then removed from the tree and transported back to camp for further processing. Measurements of the plants themselves included measuring the base circumference and the longest leaf and counting the number of leaves. Plant volume was measured as the total capacity of the bromeliad, by filling the phytotelmata with a known quantity of water until full.

### 2.3 DNA Extraction

DNA was extracted from 63 swabs using the DNeasy PowerSoil DNA kit (Qiagen) following the manufacturer's protocol with the following modification to the beginning of the protocol: as a first step, swabs were snapped off into the bead tubes provided in the kit, the C1 solution was added and samples were left to incubate at 65°C for 2 h while shaking gently. For each set of extractions, extraction controls were done using a clean swab and following the same steps as with sample extractions, as a negative extraction control. To obtain a positive control for later amplification, a swab was dipped in a ZymoBIOMICS Microbial community standard (D6300) and extracted following the same protocol as the sample extractions.

### 2.4 DNA Amplification

A subset of seven samples was amplified with qPCR to determine the sample concentration and cycle number that most evenly amplified the samples. The 10 μL qPCR reaction consisted of 5 μL SYBR Green Master Mix (Sigma‐Aldrich), 2 μL of 1 μM forward primer for the 16S rRNA subunit hypervariable V4 region (515F‐Y: 5′‐GTGYCAGCMGCCGCGGTAA‐3′; Parada et al. 2016), 2 μL of 1 μM reverse primer (806R: 5′‐GGACTACNVGGGTWTCTAAT‐3′; Apprill et al. 2015) and 1 μL of sample (4 ng/μL DNA) of 1/10, 1/20 and 1/40 dilutions. All samples were then normalised to 4 ng/μL and diluted to 1/10. The ZymoBIOMICS Microbial community standard was diluted to 1:200, which was reached by comparing amplification curves of dilutions of the standard to that of the samples, using qPCR.

Samples were amplified in plates following a design similar to that suggested by (Taberlet et al. 2012), consisting of 12 blank wells (at least one per row and column), 4 PCR negative controls, 4 positive controls extracted from the ZymoBIOMICS Microbial community standard, 4 extraction controls and 72 DNA extractions from samples. These 72 samples include least 3 replicates of each community sample. This resulted in three plates. Forward and reverse primers were tagged with phased barcodes. The PCR recipe was a 10 μL reaction consisting of 4.9 μL AmpliTaq Gold 360 (Applied Biosystems), 0.1 μL GC Enhancer (Applied Biosystems), 2 μL of 1 μM forward primer, 2 μL of 1 μM reverse primer and 1 μL of sample (0.4 ng/μL DNA). Amplification was achieved with the following programme: 10 min at 95°C, 31 cycles of 30 s at 95°C, 1 min at 50°C and 90 s at 72°C, followed by 30 min at 72°C and held at 4°C.

### 2.5 Library Preparation and Sequencing

Plates were pooled by taking 5 μL of each well from each amplified plate. Plate pools were quantified by Qubit dsDNA HS (High Sensitivity) Kit (Life technologies) for broad‐range double‐stranded DNA. Each plate pool was normalised by dilution with molecular grade water to the lowest concentration of the plate pool. Two pools were created from the three plates.

Library preparation of the two pools was performed in conjunction with a third pool of plates for another project. Library preparation was carried out using TruSeq DNA PCR‐Free Library Prep kit (Illumina) and following the manufacturer's instructions with some modifications, without the size selection following conversion of overhangs and instead purified with a 1.8:1 bead clean‐up. The TruSeq DNA Single Indexes Set A (Illumina) adapters 005, 006 and 012 were used. Library size was ascertained by an Agilent 7500 Bioanalyzer chip with the High Sensitivity DNA Kit (Agilent Technologies), from which DNA‐adapter ratios were established to estimate the concentration of the prepared libraries. We determined the size of the un‐adapted libraries and added the length of the adapters to the library size to estimate an average library length. We then calculated adapter ratios and measured the libraries with Qubit. The final library concentration was a combination of both Qubit results and the Bioanalyzer output (Hughes 2022).

Libraries were pooled to obtain 20 μL of 4 nM library. The pooled library was diluted and denatured following Illumina Dilute and Denature protocol in order to dilute the library to 20 pM and then the final dilution. The pooled library that was sequenced was diluted to 8 pM and spiked with 5% 20 pM PhiX, due to the low‐diversity of the libraries. Six hundred microlitres of library were loaded onto an MiSeq Reagent Kit v2 (Illumina) flow cell and sequenced on an Illumina MiSeq for 2 × 301 cycles, at the Manchester Metropolitan University (Manchester, UK).

### 2.6 Prokaryotic Data Processing

Paired end reads were merged using the function *illuminapairedend* in Obitools v.1.0122 (Boyer et al. 2016). Reads with an alignment score of over 50 were kept and demultiplexed using the function *ngsfilter*, which sorts reads by their barcode tag combination to their corresponding samples and removes the tag and primer sequences. The resulting data was exported using *obisplit* and imported into QIIME2 (Bolyen et al. 2019). The *Deblur* function (Amir et al. 2017) was used to denoise the data, remove chimeras and trim sequences to 252 base pairs. At this point, sequences shorter than 252 were removed along with sequences which appeared fewer than twice throughout the dataset. Denoising resulted in 3,005,775 reads. Taxonomic assignment was performed with a Naive Bayes pre‐trained SILVA 138 classifier (*silva‐138‐99‐515‐806‐nb‐classifier*) (Bokulich et al. 2018; Robeson et al. 2020), trained on the specific region of interest (515F/806R). A phylogenetic tree was constructed in order to calculate UniFrac community distances, using the *MAFFT* function in QIIME2 to align the sequences and the *FastTree* function to construct the tree, to which a midpoint was added using *phylogeny midpoint‐root*. Files were saved as QIIME2 artefacts and imported into R as a *phyloseq* object using the *qiime2R* package (Bisanz 2018). The *qza_to_phyloseq* function imports and combines the outputs from the QIIME2 pipeline: the ASV (amplicon sequence variant) or feature table, the phylogenetic tree, the taxonomic data and the information about the samples in the dataset.

ASVs were considered contaminants if they were present in the PCR and extraction controls but not in the sequencing controls or ‘blanks’ (empty wells in the PCR plates). These were therefore removed using the *prune_samples* and *prune_taxa* functions in *phyloseq* (McMurdie and Holmes 2013).

Trimming tags and primers at the same time would have eliminated most of the chimeras and artefacts caused by tag‐jump; we dealt with the remaining suspected tag‐jump following the logic of metabaR's *tagjumpslayer* (Zinger et al. 2021): the number of individual ASVs present in the sequencing controls were subtracted from those in the samples.

Functional group assignment was done using FAPROTAX (Louca et al. 2016) in Python 3.7. FAPROTAX is a Python script which assigns ASVs to functional groups based on the known functions of other members of a taxonomic group. It is therefore a rough method of functional grouping based on the functional roles of related taxa, rather than an accurate classification of an ASV's functional role, as most will be unknown. This process assigned 1319 ASVs to groups, leaving 3585 unclassified. Functional groups that could be collated into larger groups were merged (e.g., ‘methanogenesis using formate’ and ‘methanogenesis by CO_2_ reduction’ and similar were all included under the umbrella ‘methanogenesis’).

### 2.7 Statistical Analysis

All statistical analyses were performed in R 4.1.1 (R Core Team 2020). Sample replicates were merged using *merge_samples2* from *speedyseq* (McLaren 2020) and controls removed prior to analysis. Observed Richness, Shannon and Inverse Simpson indexes were calculated for each bromeliad using raw count data (McMurdie and Holmes 2014). Generalised Linear Models (GLMMs) with quasi‐poisson distribution were used to test whether each alpha diversity metric was correlated with any of the measured variables: bromeliad species; forest type; sampling season; height of the bromeliad on the tree; the volume of the bromeliad; the number of leaves; the base circumference; the longest leaf and the pH and temperature of the phytotelm. Attempts to simplify the models did not significantly improve them, therefore, all variables were retained.

Diversity index ~ height + log(volume) + pH + phytotelm temperature + longest leaf + *n*° of leaves + base circumference + forest type + bromeliad species + season, family = quasipoisson.

For beta diversity analyses, data were transformed to relative abundances. Weighted UniFrac (Lozupone et al. 2007) and Unweighted UniFrac (Lozupone and Knight 2005) distances were calculated using the *phyloseq* package in R. UniFrac distances take into account phylogenetic relationships between ASVs, with Unweighted UniFrac distances considering only presence‐absence counts while Weighted UniFrac uses abundance data as well. Permutational multivariate analyses of variance (PERMANOVA) with each distance metric were performed with the *adonis* function in the *vegan* package (Oksanen et al. 2013) with 999 permutations, to see if any of the following variables affected taxonomic composition within the bromeliads: bromeliad species, forest type, height on the tree, volume (logged), length of the longest leaf, base circumference, number of leaves, collection season and phytotelm temperature and pH. The PERMANOVAs were performed with ASV data and family data.

To see whether environmental and plant variables correlated with different functional groups, Pearson's correlations were used for the continuous variables (height, pH, phytotelm temperature, volume, longest leaf, number of leaves, base circumference), while *χ* ^2^ tests were performed for the categorical variables (forest type, season, species). The results of the Pearson's correlations and residuals [(observed − expected)/square root (expected)] of the *χ* ^2^ tests were visualised with *corrplot* (Wei and Simko 2021).

Stacked bar charts of taxomomic data were produced using the *barplot* function in *phyloseq*, showing composition in different bromeliads and in the two forest types. These were produced at the phylum and family level due to the amount of unidentified entities at the ASV level and the intractability of the number of ASVs. Stacked bar charts of functional groups were constructed in *ggplot2* (Wickham 2016).

## 3 Results

### 3.1 Plant Characteristics

Sixty‐three bromeliads (56 *A. hoppii* and 7 *A. nidularioides*) were collected. The range of plant measurements and environmental variables were as reported in O'Reilly‐Berkeley et al. (2026): phytotelmata volume ranged from 80 to 1400 mL (mean 389.36 ± 245.86 SD); longest leaf, 19–238 cm (mean 141.48 ± 40.22 cm SD); base circumference, 9–25 cm (mean 14.34 ± 3.05 cm SD); number of leaves, 8–29 cm (mean 17.98 ± 4.20 cm SD); central phytotelm pH varied from some with a strongly acidic pH 3.4 to a few phytotelmata exhibiting neutral or mildly alkaline pH 8.2 (mean 5.83 ± 0.93 SD); phytotelm temperature varied between 22.2°C and 30.0°C (mean 25.71°C ± 1.71°C SD). Heights on the tree at which bromeliads were collected ranged between 1.96 and 20.20 m above the ground (mean 7.77 ± 4.14 m SD), with 97.3% of bromeliads found above 2.5 m from the ground.

### 3.2 Total Diversity

Across 63 epiphytic bromeliads, a total of 4904 ASVs including unidentified sequences were found belonging to 49 phyla. Correcting the data for suspected tag‐jump did reduce the number of reads in the samples, but did not affect patterns of alpha‐ or beta‐diversity (Figures S2–S7).

### 3.3 Prokaryotic Alpha Diversity

Using Observed Richness, alpha diversity was not correlated with any of the measured variables. However, using Shannon and Inverse Simpson indexes, diversity was correlated with height, volume, longest leaf and sampling season, depending on which index was used (Table 1). Alpha diversity by categorical variables is plotted in Figure 1 and by continuous variables in Figures 2 and 3.

**TABLE 1:** Results of the GLMMs.

Variable	Shannon			Inverse Simpson		
	Estimate ± SE	*t*	*p*	Estimate ± SE	*t*	*p*
Height	−0.009 ± 0.004	−2.046	0.048*	−0.055 ± 0.023	−2.026	0.025*
Volume (log)	−0.056 ± 0.032	−1.753	0.085	−0.413 ± 0.159	−2.580	0.013*
Longest leaf	0.001 ± 0.000	2.312	0.025*	0.005 ± 0.002	1.757	0.084
Season (Jan–Jul)	−0.132 ± 0.047	−2.824	0.007*	−0.506 ± 0.244	−2.037	0.047*

*Note:* For simplicity, only the indexes and variables with significant results are shown. The GLMM of Observed Richness yielded no significant correlations.

* *p* < 0.05.

**FIGURE 1:**
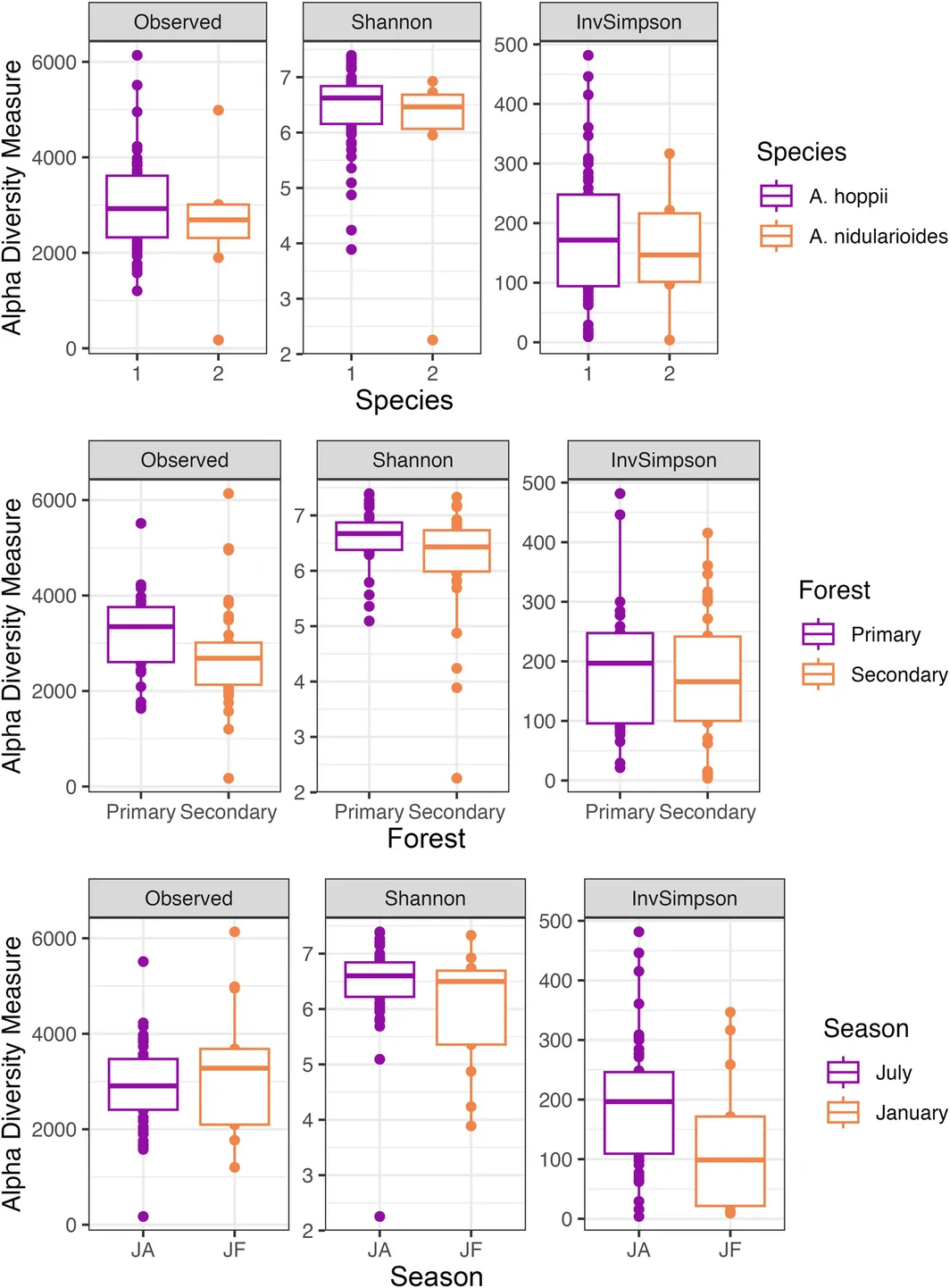
Alpha diversity as observed richness, Shannon index and Inverse Simspon index, according to categorical variables: Bromeliad species, forest type and sampling season.

**FIGURE 2:**
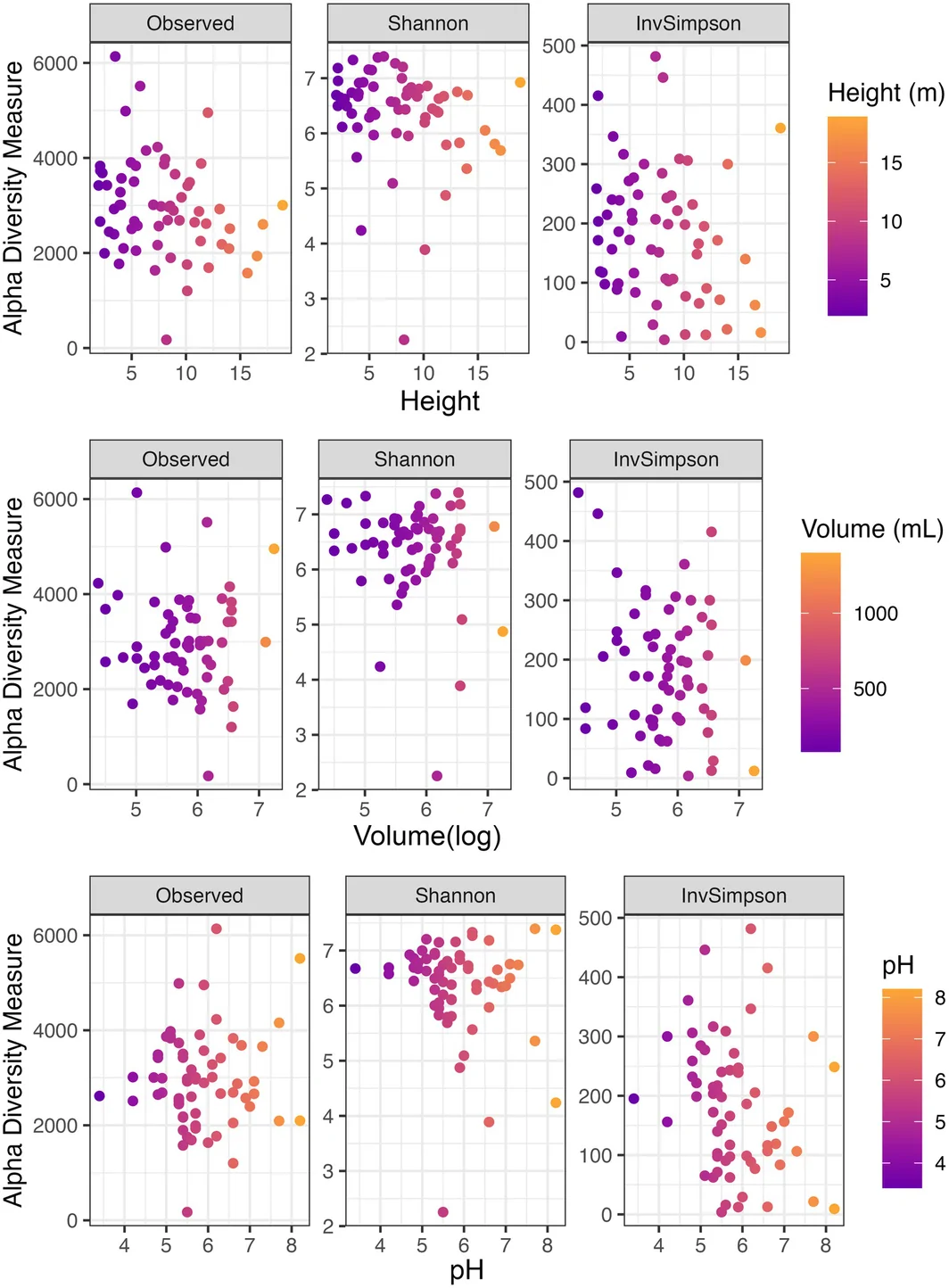
Alpha diversity as observed richness, Shannon index and Inverse Simspon index, according to base circumference, longest leaf and number of leaves.

**FIGURE 3:**
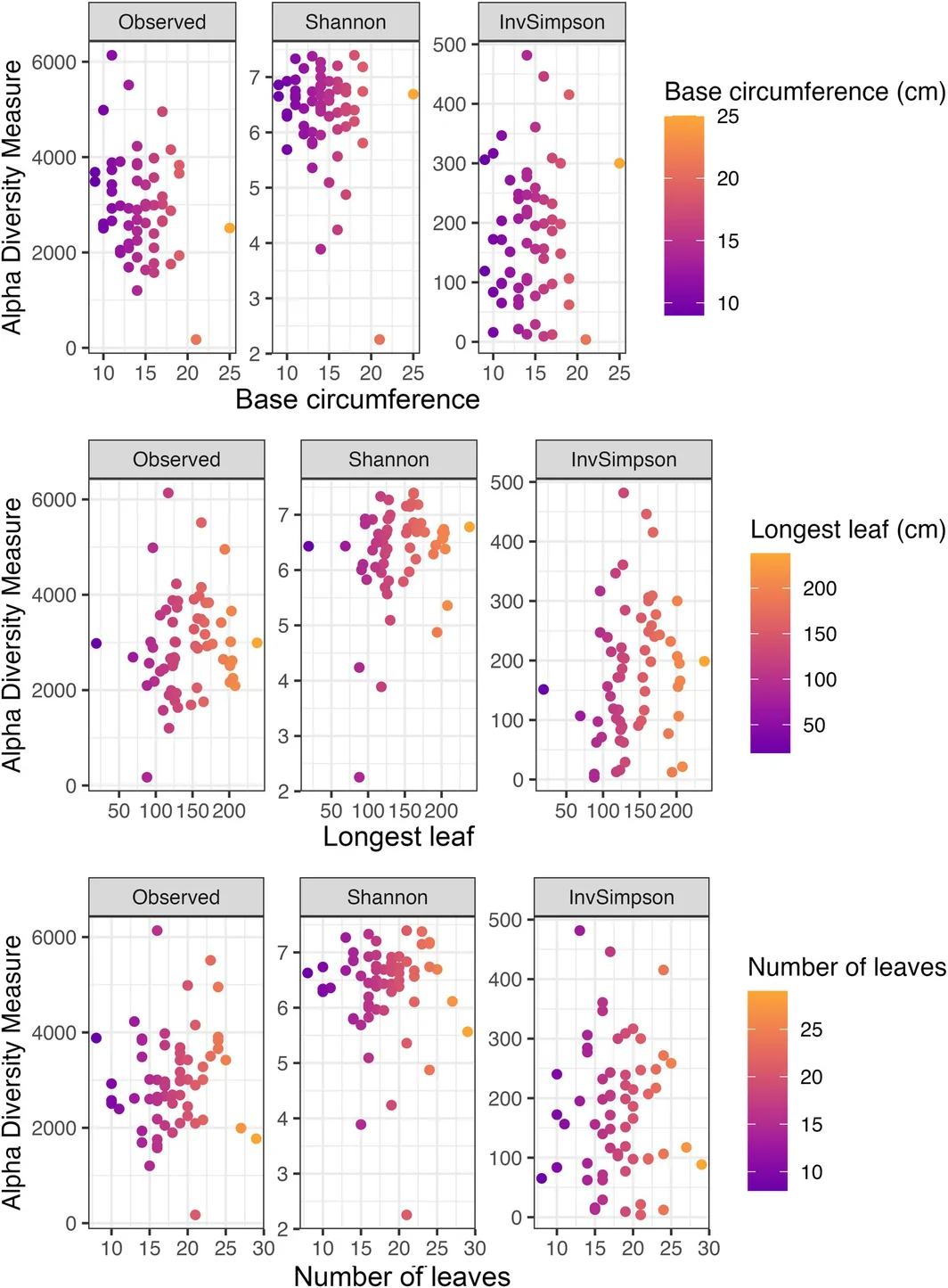
Alpha diversity as observed richness, Shannon index and Inverse Simspon index, according to height on the tree, bromeliad volume (log) and phytotelm pH.

### 3.4 Prokaryotic Beta Diversity

PERMANOVAs of both Unweighted UniFrac and Weighted UniFrac yielded very similar results at the ASV level, with height on tree and pH explaining small but significant proportions of the variation, as well as volume when Weighted UniFrac was considered (Table 2). At the family level, the same pattern occurred (Lozupone et al. 2007).

**TABLE 2:** Results of the PERMANOVAs of UniFrac distances at the ASV, family and phylum level.

		Weighted UniFrac				Unweighted UniFrac			
Taxon	Variable	df	*F*	*R* ^2^	*p*	df	*F*	*R* ^2^	*p*
ASV	Height	1, 52	1.806	0.026	0.048*	1, 52	1.399	0.021	0.018*
	pH	1, 52	2.202	0.032	0.015*	1, 52	1.434	0.022	0.012*
	Volume (log)	1, 52	1.788	0.026	0.038*	1, 52	1.030	0.014	0.630
Family	Height	1, 52	1.949	0.028	0.031*	1, 52	2.091	0.032	0.004*
	pH	1, 52	2.317	0.033	0.016*	1, 52	1.642	0.025	0.038*
	Volume (log)	1, 52	1.957	0.028	0.040*	1, 52	0.012	0.816	0.685

*Note:* Only factors which explained significant portions of variation at each level and for each distance metric are reported.

* *p* < 0.05.

We present bar charts at the family and phylum level that show the top 20 taxa at each level in order to visualise the data easily (Figure 4).

**FIGURE 4:**
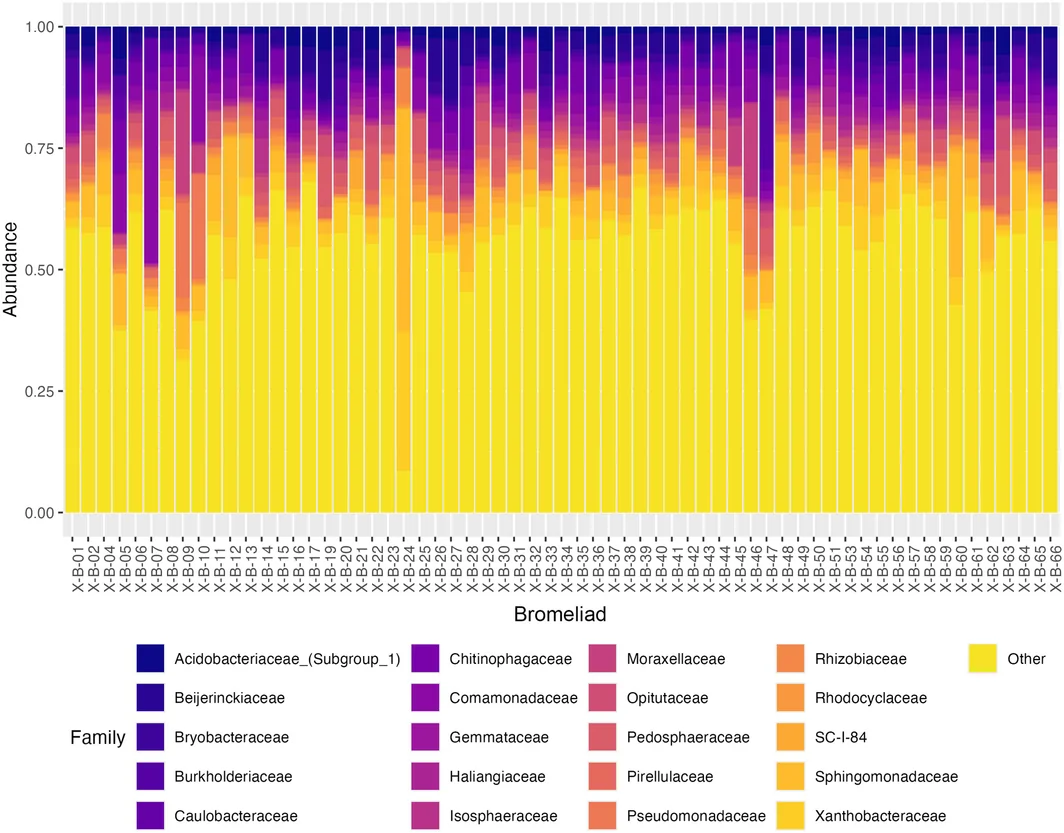
Relative abundance at the family level. Each bar represents one bromeliad sample, composed of the pool of the technical replicates.

### 3.5 Funtional Diversity

Using FAPROTAX, 1319 ASVs were successfully sorted into functional groups (Figure 5), leaving 3585 unclassified. Phytotelm pH had the highest number of associations with function, being significantly positively correlated with denitrification, nitrogen respiration, oxidation of sulphur compounds, chitinolysis, xylanolysis, fermentation, aerobic chemoheterotrophy and dark hydrogen oxidation, but was negatively correlated with cellulolysis. Height on the tree was negatively correlated with denitrification, nitrogen fixation and nitrogen respiration. Phytotelm temperature was correlated with aromatic compound degradation. Measures of plant size and complexity (volume, longest leaf, number of leaves and base circumference) were variably negatively correlated with methanogenesis, aerobic ammonia oxidation, nitrification, cellulolysis, photoheterotrophy, iron respiration, chlorate reduction and predatory or exoparasitic groups. Volume was additionally positive correlated with aerobic chemotrophy and aromatic compound degradation, while the number of leaves was positively correlated with xylanolysis (Figure 6).

**FIGURE 5:**
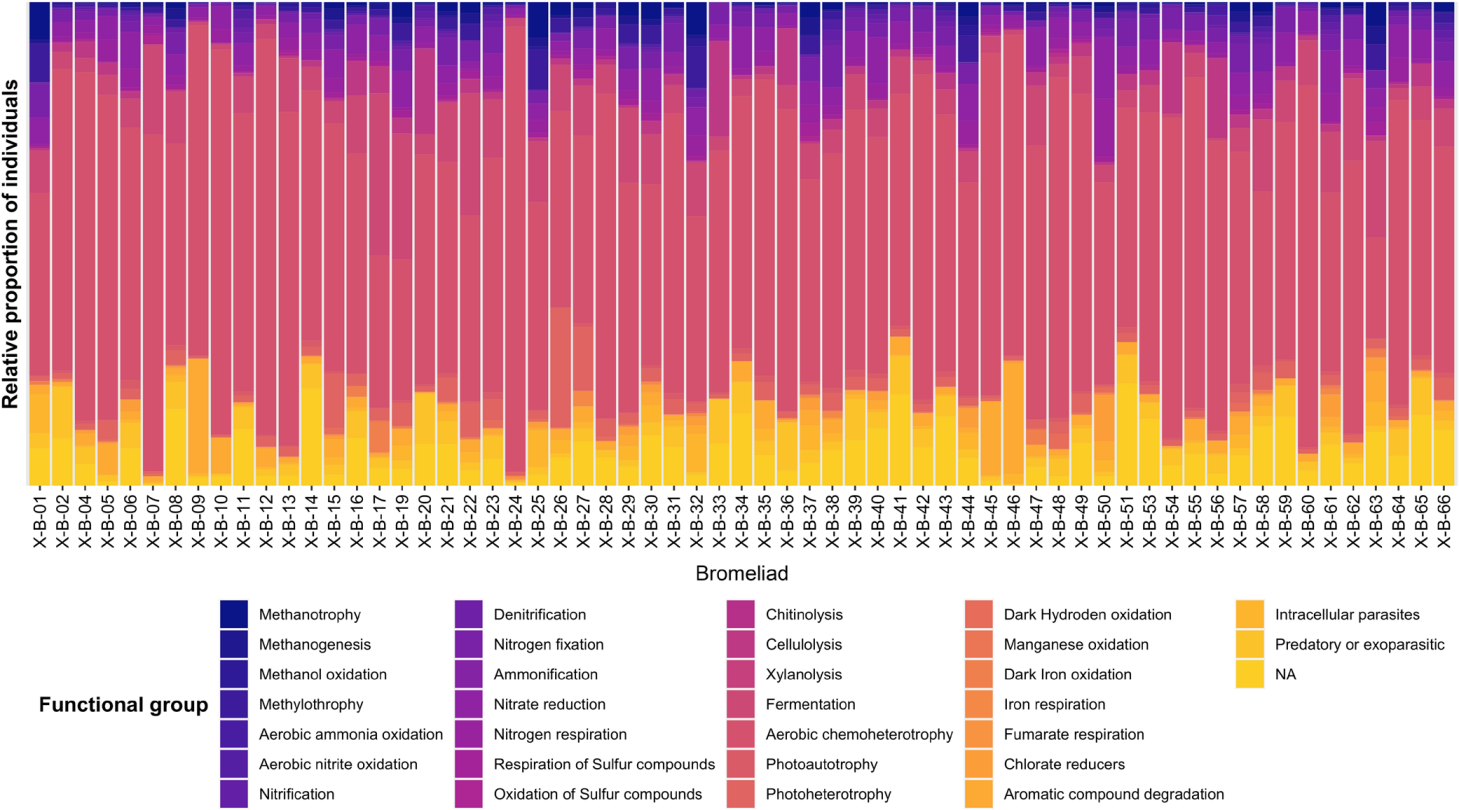
Functional group abundance in individual bromeliads.

**FIGURE 6:**
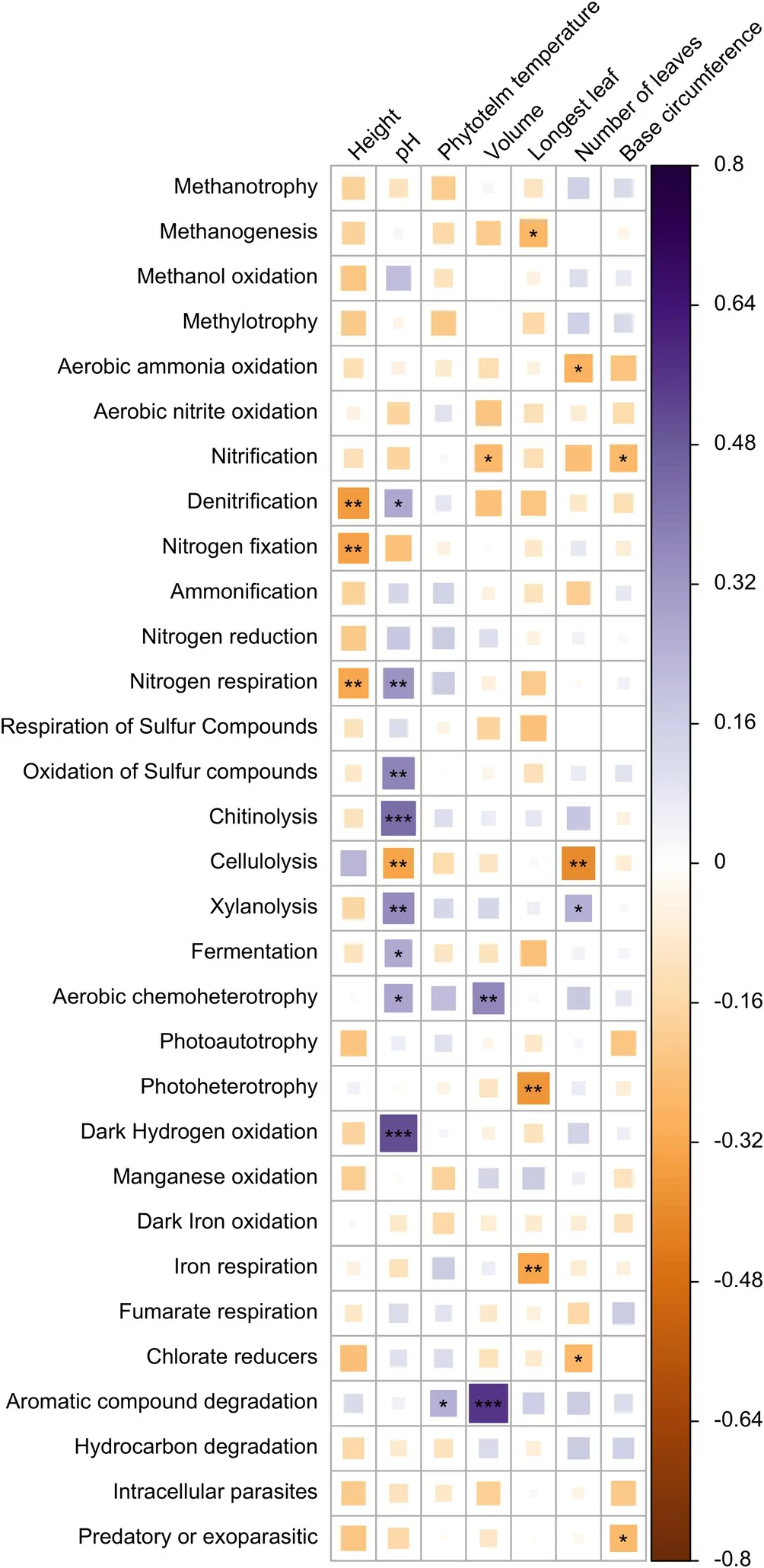
Correlation of functional groups of microbial taxa in bromeliads according to different continuous variables. Legend shows strength and direction of correlation. Asterisks show degree of significance: *0.05, **0.01, ***0.001.

Some functional groups were more strongly associated with certain a forest type, species, or sampling season. For instance, fermentation was more strongly associated with secondary forest than primary forest (Figure 7). Several group differences were found between sampling season, including nitrogen fixation, cellulolysis, aerobic chemoheterotrophy, photoheterotrophy, aromatic compound degradation and predatory or exoparasitic groups (Figure 7). Between species of bromeliad, differences between the two mostly manifested themselves as stronger affiliations or dissociations between *A. nidularioides* and certain functions, such as methanotrophy, methanogenesis, methylotrophy, nitrogen fixation, cellulolysis, fermentation, aerobic chemoheterotrophy, aromatic compound degradation, hydrocarbon degradation and predatory or exoparasitic groups (Figure 7).

**FIGURE 7:**
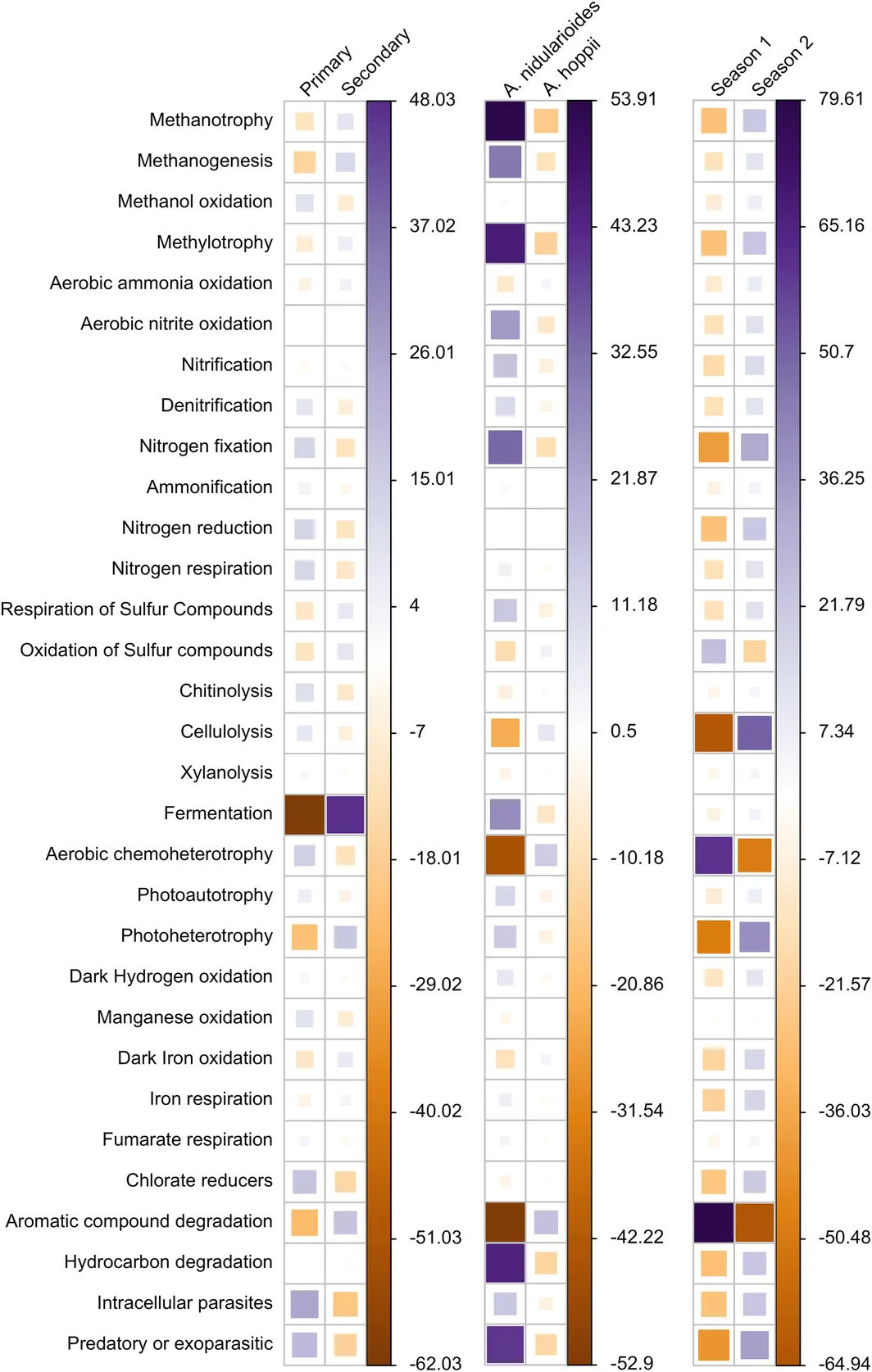
Correlation of functional groups of microbial taxa in bromeliads according to different categorical variables. Legend shows strength and direction of residuals of *χ* ^2^d test between each variable.

## 4 Discussion

Bromeliads host diverse and complex prokaryotic communities which carry out a range of functions within the phytotelm microcosm (Inselsbacher et al. 2007; Lehours et al. 2016; Leroy et al. 2016). These communities are taxonomically distinct but functionally similar to each other among bromeliads (Brandt et al. 2017; Louca, Jacques, Pires, Leal, Srivastava, et al. 2017; Simão et al. 2020). This relatively large bromeliad community metabarcoding study (in terms of sample size), focuses on bromeliads within a tropical forest and adds to our knowledge of these systems (Louca, Jacques, Pires, Leal, González, et al. 2017; Louca, Jacques, Pires, Leal, Srivastava, et al. 2017; Rodriguez‐Nuñez et al. 2018; Giongo et al. 2019; Simão et al. 2020). We found 4904 ASVs assigned to 49 Phyla across the 63 bromeliads in this study. This represents a similar number of phyla overall than seen in the most diverse study of bromeliad phytotelm communities which found 51 phyla in 16 bromeliads in a site in Puerto Rico (Rodriguez‐Nuñez et al. 2018). Although, our greater sampling size and so the average number of phyla per bromeliad is lower than in the aforementioned study, this may be due to differences in sampling methods—the aforementioned study collected phytotelm content (mostly water) while we swabbed the bottom‐most surface of the central phytotelm, thus limiting our collection to the communities of the principal tank of the bromeliads (Rodriguez‐Nuñez et al. 2018). We did this partly in an attempt to standardise our methods across bromeliads that contained very different quantities of water at the time of sampling, but in doing so may not have captured the full range of diversity present. We did not perform a comparative study to assess this although this would be interesting future work within these systems. Nonetheless, we found high diversity of prokaryotes in our sample of 63 bromeliads in a previously unexplored area, mirroring the highly diverse and complex community of bromeliad macroinvertebrates (O'Reilly‐Berkeley et al. 2026).

Several plant and habitat variables significantly influence the prokaryotic communities within their phytotelm; however, no single variable we measured explained a large portion of the variation seen, similar to the invertebrate communities sampled from the same bromeliads (O'Reilly‐Berkeley et al. 2026). Height of the bromeliad from the ground and phytotelmata volume consistently affected alpha‐ and beta‐diversity, while other variables such as pH and sampling season affected different aspects of prokaryote diversity. In terms of functional diversity, a complex picture of correlations emerged, where different variables correlate with different functional groups (Figures 6 and 7).

### 4.1 Effect of Height From the Ground

The height from the ground at which bromeliads were attached to their host tree significantly affected alpha‐diversity (as measured by Shannon and Inverse Simpson indices, but not observed richness) and the community composition of prokaryotes in the phytotelm. Alpha diversity was negatively correlated with height, suggesting as one ascends into the canopy the prokaryotic community is less diverse. A previous study found no effect of height on bacterial density in bromeliads (Brouard et al. 2012), but they only looked at bromeliads up to 2 m from the ground, whereas our sampling incorporated individuals up to 20 m. However, other studies of canopy microbiomes have found decreasing levels of alpha diversity as well as differences in community composition in the phyllosphere depending on position in the canopy, as seen within the bromeliad phytotelmata here (Izuno et al. 2016; Stone and Jackson 2019; Herrmann et al. 2021). Although bromeliads form canopy oases with different conditions to their surrounding environment, it appears that some patterns in microbial diversity follow general patterns of canopy phyllosphere microbial communities. In the case of other phyllosphere communities, richness may decrease with increasing height due to the more extreme conditions (higher exposure to UV, heat, wind, rain) of the canopy (Stone and Jackson 2019). Although none of the bromeliads sampled here were exposed to the extreme conditions of the upper canopy, it would be interesting to measure vertical variation in microclimatic conditions to ascertain whether it is environmental conditions or biotic factors which dictate this pattern. Additionally, we did not measure debris or leaf litter content of the bromeliads, which could vary ascending into the canopy, consequently affecting the microbial communities (Carrias et al. 2020).

The same bromeliads displayed no variation with height of macroinvertebrate richness (O'Reilly‐Berkeley et al. 2026), however community composition did vary vertically and these communities are known to be trophically‐linked through complex interactions (Céréghino et al. 2020). This was true whether abundance or presence‐absence data was analysed, suggesting vertical variation in both aspects of community composition. Height on the tree has been shown to affect invertebrate communities in bromeliads in some cases (Brouard et al. 2012; Kratina et al. 2017). Although to our knowledge there has not been an association drawn between bacterial densities and height of the bromeliad on the tree (Brouard et al. 2012), we detected an effect of height on communities at both of the analysed taxonomic levels, suggesting previous non‐molecular studies may have failed to detect subtle differences in community composition. Certainly, studies of other phyllosphere systems in the canopy have shown that position in the canopy can be a stronger determinant of bacterial community composition than other facts such as rain disturbance or (sometimes) tree species identity (Izuno et al. 2016; Laforest‐Lapointe et al. 2016; Stone and Jackson 2019; Herrmann et al. 2021). As with alpha‐diversity, it would be informative to measure microclimate conditions across the complete vertical gradient at which bromeliads are found.

Height was negatively correlated with functional groups of bacteria involved in denitrification or nitrogen respiration (the reduction of nitrate into molecular dinitrogen, through a series of other forms) and nitrogen fixation (i.e., the conversion of molecular dinitrogen into ammonia). This implies differences in nitrogen cycling throughout the vertical gradient, with less activity as one ascends into the canopy. This could be related to the overall reduction in ASV richness with increasing height. With increasing height, light availability can increase and with it the prevalence of photoautotrophs, which could compete with certain prokaryotic groups, although we can only hypothesise because we did not sample algae or measure canopy cover.

### 4.2 Effect of Bromeliad Size

As structurally complex structures, bromeliad size can be measured in different ways. The length and shape of the leaves, the number of leaves, the base circumference, the number and depth of phytotelmata and the volume of phytotelmata can all be considered measures of size, although the number of leaves can additionally be used as a measure of habitat complexity. We measured the length of the longest leaf, the base circumference and the potential volume and counted the number of leaves in bromeliads and found they were not correlated (O'Reilly‐Berkeley et al. 2026), therefore, we analysed all four variables.

The length of the longest leave was weakly positively correlated with the Shannon index but not other alpha‐diversity indeces, suggesting that the signal is not a strong one, although it could plausibly be related to an unmeasured variable affecting both the length of leaves and the prokaryotic community, such as irradiation or plant age. The only other proxy for size which correlated with alpha‐diversity (Inverse Simpson index), was phytotelm volume. However, surprisingly this correlation was negative, whereas the opposite tends to be true for bromeliad invertebrate communities (Richardson 1999; Jocque and Field 2014; Srivastava, Ware, et al. 2020) and other microbial communities (Bell et al. 2005; Delgado‐Baquerizo, Eldridge, et al. 2018), where larger plants host more diverse communities. Again however, this correlation was weak and only significant using one diversity index and so may be artifactual.

Volume was also the only size measurement to affect community composition of prokaryotes in bromeliads. At the ASV level, although it explained only 2.6% of observed variation in the community, volume significantly affected community composition using Weighted UniFrac distances, suggesting that part of the changes in the community composition due to volume are related to differential abundances of taxa rather than differences in the presence or absence of taxa (as would be expected if the results were significant when the Unweighted UniFrac distances were considered). This may be related to the higher colonisation surface allowing for different abundances of taxa to inhabit the tank even when a similar threshold of prokaryotic richness has been reached. Madsen (2020) reported an effect of maximum rock pool size on the community composition of bacteria. In this case, similarly to here with our bromeliads, volume only explained a small proportion of the variation (2%) (Madsen 2020). However, such a small amount of variation may not be ecologically significant.

Aerobic chemoheterotrophy and aromatic compound degradation significantly increased with increasing phytotelm volume, while nitrification decreased albeit to a lesser extent. This suggests that more organic decomposition occurs in bigger bromeliads. The effect of bromeliad capacity on leaf litter decomposition is context‐dependent, having been shown to differ depending on site through effects of bromeliad size on the invertebrate community (LeCraw et al. 2017). Larger bromeliads may also collect more leaf litter, driving the increase in decomposition of organic matter (Aguilar‐Cruz et al. 2022).

### 4.3 Effect of pH

The pH of the environment affects bacterial community composition in a range of different habitats, from soil (Zhalnina et al. 2015; Yun et al. 2016) to gut communities (O'May et al. 2005; Duncan et al. 2009), with different taxa tolerating different levels of acidity. In freshwater bodies, pH is known to affect prokaryotic composition and diversity (Lindström et al. 2005; Banda et al. 2021), we therefore were surprised to find pH did not affect the alpha diversity of our sample as it has in previous research on bromeliads (Goffredi, Kantor, et al. 2011). However, it did affect community composition and functional diversity of the prokaryotic community.

The sampled bromeliads had a wide range of pH values, from a strongly acidic pH 3.4 with a few with a more neutral or mildly alkaline pH 8.2, although the mean from all sampled bromeliads was acidic, at 5.83 (±0.93 SD). This steep pH gradient within our sample could be due partly to the varying degrees of water levels in the tanks, as well as different stages of leaf litter decomposition. The taxonomic variation with pH was found to be reflected in correlations between pH and particular functional types, with certain groups increasing with higher pH values, such as those involved in denitrification and nitrogen respiration, oxidation of sulphur compounds, chitinolysis, xylanolysis and aerobic chemoheterotrophy. In contrast, cellulolysis activity increased with lower pH values, which is surprising as acidic pH has been associated with lower cellulolytic activity in other decompositional systems (Li et al. 2026). Despite these trends, most of these processes occur most efficiently at medium values of pH when bacteria studied as isolates (González‐Sánchez and Revah 2007; Saleh‐lakha et al. 2009; Wahyuntari et al. 2009; Kielak et al. 2013; Delgado‐Baquerizo, Oliverio, et al. 2018; Qian et al. 2019), closer to the mean pH within our sample.

### 4.4 Effect of Sampling Season

The study site where we collected the bromeliads lies in an area with very little seasonal variation (Irvine 1987; personal communications with members of the local community). Despite this, we found that communities differed in richness during our two sampling campaigns: January–February 2018 and July–August 2018. We did not measure precipitation or temperature variation during this time and so it is not possible to link these patterns to atmospheric conditions. It is entirely possible that such variation could have driven differences between prokaryotic communities in the bromeliads as precipitation does other components of these communities (Buosi et al. 2015; Srivastava, Céréghino, et al. 2020), however, without repeated annual data we cannot say if these patterns are seasonal or an artefact of either different atmospheric conditions or even biological seasonality in the forest (Stafford et al. 2016). There are indications that conditions (such as nutrient availability) within phytotelmata vary throughout the year (Giongo et al. 2019), which could either drive or be a consequence of prokaryotic community dynamics. However, it would be interesting to sample the same bromeliads throughout the year in order to ascertain if there is truly seasonality in the prokaryotic community within bromeliad phytotelmata in this region.

## 5 Conclusion

We present a broad and preliminary picture of the prokaryotic diversity inside epiphytic bromeliads in one of the most biodiverse regions on Earth. As we expected, height on the tree, pH and volume of the phytotelm affected the prokaryotic community composition, but sampling season affected ASV richness in the bromeliads. However, these factors did not explain much of the community variation. More work is needed to understand these patterns in bromeliads, particularly what drives changes in community composition as one ascends towards the canopy, which could involve measuring microclimatic variation along the vertical gradient. Additionally, further work is needed to understand the mechanisms through which the measured variables affect functional diversity. Overall, we present an initial but complex picture of how a selection of habitat and plant characteristics influence the prokaryotic communities in epiphytic bromeliads, providing a baseline upon which to explore further questions.

## Author Contributions

**Xaali O'Reilly‐Berkeley:** conceptualization, methodology, investigation, data curation, formal analysis, writing – review and editing, writing – original draft, visualization, project administration. **Thomas D. Hughes:** methodology, formal analysis, writing – review and editing. **Hannah Mossman:** writing – review and editing, supervision. **Jennifer K. Rowntree:** writing – review and editing, supervision, project administration. **Richard F. Preziosi:** conceptualization, investigation, supervision, writing – review and editing, resources.

## Funding

This work was supported by the Manchester Metropolitan University through a PhD programme, and the Genetics Society (UK) through a Hereditary Fieldwork Grant.

## Ethics Statement

This work was carried out while all authors worked at the Manchester Metropolitan University. The field work for this project was carried out with permission from the ministry of the environment of Ecuador (Ministerio del Ambiente, MAE) and in collaboration with the Ecuadorian national biodiversity institute (Instituto Nacional de Biodiversidad, INABIO), under the research permit number MAE‐DNB‐CM‐2016‐0045. This work was carried out in accordance with the formal agreement between the San José de Payamino Community and the Timburi Cocha Research Station.

## Conflicts of Interest

The authors declare no conflicts of interest.

## Supporting information


**Table S1:** Microsatellite primer sequences, allele repeat motifs and size ranges, with tail‐dye combinations. BC = ‘Tail C’ (Blacket et al. 2012), BB = ‘Tail B’ (Blacket et al. 2012), M13B = ‘M13modB’ (Culley et al. 2013).
**Figure S1:** NMDS using Rogers' genetic distance.
**Figure S2:** Total reads per sample before and after correction for suspected tag‐jump.
**Figure S3:** Observed richness before and after tag‐jump correction.
**Figure S4:** Shannon index before and after tag‐jump correction.
**Figure S5:** Inverse Simpson index before and after tag‐jump correction.
**Figure S6:** NMDS comparison of tag‐jump correction using Unweighted UniFrac distances.
**Figure S7:** NMDS comparison of tag‐jump correction using Weighted UniFrac distances.

## Data Availability

The sequencing data is available on the ENA (European Nucleotide Archive, www.ebi.ac.uk/ena) under project ERP182389 and Dryad at https://doi.org/10.5061/dryad.sf7m0cgm5.
