# Supplementary material for: Height From the Ground and Phytotelm pH Affect Prokaryotic Diversity in Epiphytic Bromeliads
Source: Environ Microbiol Rep. 2026 Oct 22;18(5):e70421. doi: 10.1111/1758-2229.70421 (PMC13687927; doi:10.1111/1758-2229.70421)
Supplement: Supplementary file 1 [file EMI4-18-e70421-s001.docx]

Habitat and plant characteristics affect prokaryotic diversity in bromeliad phytotelmata

**Supplementary Information**

# 1. Microsatellite development and plant genotyping

Bromeliads were genotyped using microsatellite markers developed specifically for *A. hoppii* and *A. nidularioides*, following Fox et al. (2019) and Griffiths et al. (2016). In order to separate the two species, microsatellite markers were developed which cross-amplified in *A. hoppii* and *A. nidularioides*. Marker development followed the Multi-individual Microsatellite identification (MiMi) method described in Fox et al. (2019). DNA of eight individuals of flowering bromeliads (four *A. hoppii* and four *A. nidularioides*) was extracted and library preparation was performed using the Nextera® XT Library Prep Kit (Illumina) following the manufacturer’s instructions.

Initial microsatellite identification for each bromeliad was carried within the Galaxy Centaurus server of the University of Manchester’s Core Bioinformatics Facility, following Griffiths *et al.* (2016). Trimmomatic (Bolger, Lohse and Usadel, 2014) within Pal_filter (Griffiths *et al.*, 2016) was used for quality control and trimming reads, with the following settings: sliding window size = 4bp, quality = 20, leading = 3, trailing = 3, minlen = 50. Reads were paired using PANDAseq (Masella *et al.*, 2012) and potentially amplifiable microsatellite loci were identified using Pal_finder (Castoe *et al.*, 2012). Primers for potentially amplifiable loci were designed in Pal_finder using Primer3 (Untergasser *et al.*, 2012), with the following parameters: primer_opt_size = 21, primer_min_size = 20, primer_max_size=30, primer_min_GC = 40.0, primer_max_GC = 60.0, primer_min_TM = 59.0, primer_max_TM = 68.0, primer_opt_TM = 60.0. These parameters were set so that downstream, primers could be amplified using the Type-It® Microsatellite Kit (Qiagen).

Identified primer regions for each of the eight individuals were then compared using MiMi (Fox *et al.*, 2019). MiMi was run separately for each bromeliad species, using the same terms. MiMi was set to find primers of polymorphic loci found in at least 50% of individuals.

The MiMi pipeline identified 91 and 65 potential microsatellite markers fitting the specified parameters in *A. hoppii* and *A. nidularioides,* respectively, and filtered 12 and 13, respectively, as being of high quality. All 25 of these markers amplified in both species of bromeliad. PCR conditions for testing markers were as follows: 5 minutes at 95ºC, 35 cycles of 30 seconds at 95ºC, 90 seconds at 60ºC, 30 seconds at 72ºC, followed by 30 minutes at 60ºC. A universal “tail” sequence was added to the 5’ end of the forward primer of successful primer pairs, in order to cost-effectively label the primers with fluorophores according to Culley *et al.* (2013) and Blacket *et al.* (2012). The labelled forward primer was then included in the reaction as a third primer (Culley *et al.*, 2013).The fluorophores added to the tail primers were either 6 FAM, HEX, or ROX, added to the tails “Tail C” (CAGGACCAGGCTACCGTG) (Blacket *et al.*, 2012), “Tail B” (GCCTTGCCAGCCCGC) (Blacket *et al.*, 2012), or “M13modB” (CACTGCTTAGAGCGATGC) (Culley *et al.*, 2013), respectively. Markers were tested with fluorophores and primers which appeared to amplify fewer than 5/8 of samples (absence of bands on an agarose gel) were excluded from further testing.

Twelve markers were selected for Fragment Length Analysis, three of which were thereafter excluded due to producing several alleles per locus in one case and little variation in the others; a further marker was excluded due to linkage disequilibrium. Eight markers were used in the final genotyping.

Supplementary Information Table 1. Microsatellite primer sequences, allele repeat motifs and size ranges, with tail-dye combinations. BC = “Tail C” (Blacket et al., 2012), BB = “Tail B” (Blacket et al., 2012), M13B = “M13modB” (Culley et al., 2013).

| Primer | Forward sequence | Reverse sequence | Motif | Size range | Tail -dye |
| --- | --- | --- | --- | --- | --- |
| AH1 | TCAATACCGTCACCA ATAGCC | TTCCCCTCTATTAGC CATTCC | AG | 311-365 | BC - 6FAM |
| AH5 | CAAATTGTGAATGCG AGAAGG | CTCAATCGGACATT CCAAAGG | AC | 350-365 | BC - 6FAM |
| AH9 | GAAAGAAATTGACAA CCGTGC | CTTTGTTGTCAAAG GTGATTGC | AT | 349-453 | BB - HEX |
| AN5 | TCTTTCTCACTTTTCTC CCCG | TAGATGGCACTGAT GATGACG | TC | 220-337 | M13B - ROX |
| AN7 | GCGATTCCAACTATTT TGACG | CAGTTTCCACTCTG CGAGC | TC | 399-420 | M13B - ROX |
| AN10 | TATCGGACTCCTCTCT CCTGC | AAGAAGCGCACACT AAAACCC | TC | 175-184 | M13B - ROX |
| AN12 | TCCTCCACTATGCCTC TACCG | GGCATAGAAGAAA  AGTAGCATAAAGGG | TCG | 147-191 | M13B - ROX |
| AN13 | CCTCAACGATTGCCTT TGC | CTCGGCGTTACCAT  TAGATCG | TTC | 212-237 | M13B - ROX |

Amplification of eight microsatellite regions was performed in singleplexes for each of the eight markers in 5 µl reactions, using the Type-It® Microsatellite Kit (Qiagen) under the following conditions: 5 minutes at 95ºC, 35 cycles of 30 seconds at 95ºC, 90 seconds at 60ºC, 30 seconds at 72ºC, followed by 30 minutes at 60ºC. Following amplification, 2 µl of water was added to the PCR amplicons. Plates were sent to the DNA Sequencing & Services Facility of the University of Dundee (Dundee, UK), where 0.5 µl of amplicon was prepared with 0.5 µl LIZ500 size standard (Thermo-Fisher Scientific) and 9 µl HiDi Formamide (Thermo Fisher Scientific). Amplicons were sized using a 3730 DNA analyser (Applied Biosystems). Alleles were then scored using *Fragman* (Covarrubias-Pazaran *et al.*, 2016) and bins were established and checked using *MsatAllele* v1.05 (Alberto, 2009) in R v4.1.1 (R Core Team 2020).

# 2. Plant genetic analysis

All genetic analyses were performed in R v4.1.1 (R Core Team 2020). Markers were checked for linkage disequilibrium using the *GENEPOP* package v4.6 (Raymond and Rousset, 1995; Rousset, 2008). Due to linkage disequilibrium between markers AH2 and AN5, AH2 was removed from further analysis.

Clusters of genetically similar samples were identified using the *find.clusters* function of the *adegenet* package v2.1.5 (Jombart, 2008). This function uses a Bayesian Information Criterion (BIC) to compare different clustering solutions for an algorithm which analyses the variance between potential clustering groups. The number of two clusters was selected to use for further analysis, based on the BIC not lowering substantially beyond this value (Jombart, Devillard and Balloux, 2010). Discriminant Analysis of Principal Components (DAPC) was used to assess the relationship between the two clusters (Jombart, Devillard and Balloux, 2010). Rogers’ genetic distances were calculated between bromeliads using eight of the microsatellite markers in *adegenet* (Rogers, 1972; Jombart, 2008). Non-metric multidimensional scaling (NMDS) was constructed using the *vegan* v2.5.7 package (Oksanen *et al.*, 2020), based on the Rogers’ genetics distances. Two clear clusters were visible in the NMDS plot (Supplementary Information Figure 1). One cluster included all the known *A. nidularioides* individuals based on inflorescence presence. We thus split the data by cluster in the analysis, although cluster did not explain significant variation in the data.

Supplementary Information figure 1. NMDS using Rogers' genetic distance.

# 3. Effect of removing suspected remaining tag-jump on data

Most artefacts consequence of tag-jump are assumed to have been removed along with the trimming of primers and tags descbribed in Methods. However, to ensure tag-jump would not bias our results, the number of individual ASVs present in the blank sequencing controls were subtracted from those in the samples, using a function following *metabaR*'s *tag-jumpslayer* (Zinger *et al.*, 2021).

We compared alpha and beta diversity of the communities before and after extra tag-jump removal. Differences were very slight and not significant (see Supplementary Information Figures 2-7). However, we kept this filtering step regardless and they slightly changed which factors explained statistically significant portions of the patterns in the prokaryotic communities.

Supplementary Information figure 2. Total reads per sample before and after correction for suspected tag-jump.

Supplementary Information Figure 3. Observed richness before and after tag-jump correction.

Supplementary Information figure 4. Shannon index before and after tag-jump correction.

Supplementary Information figure 5. Inverse Simpson index before and after tag-jump correction.

Supplementary Information figure 6. NMDS comparison of tag-jump correction using Unweighted UniFrac distances.

Supplementary Information figure 7. NMDS comparison of tag-jump correction using Weighted UniFrac distances.

# 4. Supplementary Information references

Alberto, F. (2009) ‘MsatAllele-1.0: An R package to visualize the binning of microsatellite alleles’, Journal of Heredity, 100(3), pp. 394–397. doi: 10.1093/jhered/esn110.

Blacket, M. J. et al. (2012) ‘Universal primers for fluorescent labelling of PCR fragments-an efficient and cost-effective approach to genotyping by fluorescence’, Molecular Ecology Resources, 12(3), pp. 456–463. doi: 10.1111/j.1755-0998.2011.03104.x.

Bolger, A., Lohse, M. and Usadel, B. (2014) ‘Trimmomatic: A flexible trimmer for Illumina sequence data’, Bioinformatics, 30(15), pp. 2114–2120.

Castoe, T. A. et al. (2012) ‘Rapid microsatellite identification from illumina paired-end genomic sequencing in two birds and a snake’, PLoS ONE, 7(2). doi: 10.1371/journal.pone.0030953.

Covarrubias-Pazaran, G. et al. (2016) ‘Fragman: An R package for fragment analysis’, BMC Genetics, 17(1), pp. 1–8. doi: 10.1186/s12863-016-0365-6.

Culley, T. M. et al. (2013) ‘ An Efficient Technique for Primer Development and Application that Integrates Fluorescent Labeling and Multiplex PCR ’, Applications in Plant Sciences, 1(10), p. 1300027. doi: 10.3732/apps.1300027.

Fox, G. et al. (2019) ‘Multi-individual microsatellite identification: A multiple genome approach to microsatellite design (MiMi)’, Molecular Ecology Resources, 19(6), pp. 1672–1680. doi: 10.1111/1755-0998.13065.

Griffiths, S. M. et al. (2016) ‘A Galaxy-based bioinformatics pipeline for optimised, streamlined microsatellite development from Illumina next-generation sequencing data’, Conservation Genetics Resources, 8(4), pp. 481–486. doi: 10.1007/s12686-016-0570-7.

Jombart, T. (2008) ‘Adegenet: A R package for the multivariate analysis of genetic markers’, Bioinformatics, 24(11), pp. 1403–1405. doi: 10.1093/bioinformatics/btn129.

Jombart, T., Devillard, S. and Balloux, F. (2010) ‘Discriminant analysis of principal components: a new method for the analysis of genetically structured populations’, BMC Genetics, 11(94).

Masella, A. P. et al. (2012) ‘PANDAseq : PAired-eND Assembler for Illumina sequences’, BMC Bioinformatics, 13(1), p. 31. doi: 10.1186/1471-2105-13-31.

Oksanen, J. et al. (2020) ‘vegan: Community Ecology Package. R package version 2.5-7. 2020’.

R Core Team (2020) ‘R: A language and environment for statistical computing’. Vienna, Austria: R Foundation for Statistical Computing. Available at: http://www.r-project.org.

Raymond, M. and Rousset, F. (1995) ‘GENEPOP (version 3.3): population genetics software for exact tests and ecumenicism’, Journal of Heredity, 86, pp. 248–249.

Rogers, J. S. (1972) ‘Measures of genetic similarity and genetic distances’, in Studies in Genetics VII. University of Texas Publication, pp. 145–153.

Rousset, F. (2008) ‘GENEPOP’007: A complete re-implementation of the GENEPOP software for Windows and Linux’, Molecular Ecology Resources, 8(1), pp. 103–106. doi: 10.1111/j.1471-8286.2007.01931.x.

Untergasser, A. et al. (2012) ‘Primer3-new capabilities and interfaces’, Nucleic Acids Research, 40(15), pp. 1–12. doi: 10.1093/nar/gks596.

Zinger, L. et al. (2021) ‘metabaR: An r package for the evaluation and improvement of DNA metabarcoding data quality’, Methods in Ecology and Evolution, 12(4), pp. 586–592. doi: 10.1111/2041-210X.13552.
